# Evaluating a Community-Based Reablement Program in Taiwan: A Cluster RCT and Using the RE-AIM Framework

**DOI:** 10.1093/geroni/igaf122.2447

**Published:** 2025-12-31

**Authors:** Hsiao-Wei Yu, Yen-Po Yeh, Ya-Mei Chen

**Affiliations:** National Taiwan University, Taipei, Taipei, Taiwan (Republic of China); Changhua County Public Health Bureau, Changhua City, Changhua County, Taiwan (Republic of China); National Taiwan University, National Taiwan University, Taipei, Taiwan (Republic of China)

## Abstract

This study introduces a community-based reablement program implemented at the county level in central Taiwan using a cluster randomized controlled trial. The study has two objectives: (1) to evaluate the efficacy of the pilot program and (2) to explore its scalability using the RE-AIM framework. The pilot trial recruited 231 dyads of older adults and their family caregivers from nine adult day centers. The experimental group received a three-month intensive individualized and group-based reablement training delivered by a collaborative team of therapists and care assistants, while the control group received only group-based training. Preliminary findings indicate significant improvements in the number of hours spent performing daily activities and slight benefits in balance among participants in the experimental group compared to the control group. However, no significant changes were observed in ADLs, IADLs, cognitive function, caregiver burden, or mastery. The RE-AIM framework provides a structured approach to assess the program’s generalizability and long-term feasibility. Most staff involved in the program expressed positive attitudes toward reablement training, emphasizing its potential to enhance independence. Implementation fidelity was supported by a standardized training protocol and well-trained therapists and care assistants. Additionally, outer setting consultants played a crucial role in maintaining fidelity and providing guidance to adult day centers. However, challenges remain, including the need for sustained funding and continuous incentives to support program expansion. The increased workload among care assistants delivering reablement training also highlights the importance of resource allocation and workforce support for successful integration into routine care.

